# Screening Tools for the Early Identification of Palliative Care Needs in Patients with Advanced Chronic Conditions: An Updated Systematic Review

**DOI:** 10.3390/jcm15030919

**Published:** 2026-01-23

**Authors:** Ana Bustamante-Fermosel, Agustín Diego Chacón-Moreno, Laetitia Hennekinne, Fuensanta Gil-Gil, Helena Notario-Leo, Ricardo Larrainzar-Garijo, Juan Torres-Macho, Anabel Franco-Moreno, Gerardo García Melcón, on behalf of the Research in Palliative Care HUIL-Group

**Affiliations:** 1 Palliative Care, Hospital Universitario Infanta Leonor-Virgen de la Torre, 28031 Madrid, Spain; 2 Internal Medicine Department, Hospital Universitario Infanta Leonor-Virgen de la Torre, 28031 Madrid, Spain; 3 Faculty of Medicine, Universidad Complutense de Madrid, 28040 Madrid, Spain; rlarrain@ucm.es; 4 Emergency Department, Palliative Care, Hospital Universitario de Guadalajara, 19002 Guadalajara, Spain; 5 Hospital Universitario Infanta Leonor-Virgen de la Torre, 28031 Madrid, Spain

**Keywords:** artificial intelligence, chronic conditions, early detection, machine learning, needs assessment, palliative care, screening tools, systematic review

## Abstract

**Background/Objectives**: Earlier initiation of palliative care improves clinical outcomes, including better symptom relief, enhanced quality of life, and decreased use of healthcare resources in advanced disease. This systematic review aimed to identify and critically appraise existing tools, both conventionally developed and based on artificial intelligence, designed to identify patients eligible for early palliative care interventions. **Methods**: Six electronic databases were examined for primary research studies published between 2000 and 2025. Studies that described or evaluated screening instruments developed to support the early identification of adult patients with palliative care needs underwent dual reviewer screening and data extraction. **Results**: A total of 35 studies were included. Of these, 13 reported the development of screening tools and 22 focused on the external validation of these instruments. Nine tools were developed using traditional methods, and four instruments were created using artificial intelligence techniques. Significant heterogeneity was observed in tool design and target populations. Most screening tools used death prediction as a proxy, with limited integration of psychological and spiritual dimensions. External validation studies primarily focused on predicting mortality. Overall, all the tools showed moderate predictive ability. **Conclusions**: The ability of current screening tools to identify patients with advanced diseases who are likely to have palliative care needs remains limited. Further research is needed to develop standardized screening processes that address not only mortality prediction but also disease trajectory and functional decline.

## 1. Introduction

The rising prevalence of chronic diseases and population aging has driven a paradigm shift in global health, highlighting the need for a comprehensive re-evaluation of healthcare models. Millions of individuals live with advanced chronic conditions, both oncological and non-oncological, which substantially affect multiple dimensions of health and require a coordinated, multidimensional response [1].

Palliative care (PC) has emerged as a key component of person-centered care, addressing the complex needs of patients with life-threatening illnesses [2]. It is a comprehensive care model designed to enhance quality of life through interdisciplinary teams that manage physical symptoms, pain, and psychosocial and spiritual concerns. Furthermore, PC emphasizes the integration of patient’s support network, including caregivers and family members, who also face the emotional and clinical challenges associated with caring for these patients.

Extensive evidence supports the beneficial impact of PC, particularly in patients with advanced oncological diseases [3,4,5,6]. It not only improves quality of life and physical and psychological well-being but also reduces anxiety, depression, and caregiver burden. Notably, early integration of outpatient PC has been associated with improved survival rates, emphasizing the importance of incorporating PC at the initial stages of treatment for patients with advanced cancer. Although much of the evidence has focused on oncological populations, emerging studies suggest that PC can significantly improve outcomes for patients with non-oncological conditions, highlighting the need for broader application [7,8,9,10].

The World Health Organization (WHO) estimates that approximately 56.8 million people require PC [11]. This need encompasses patients with advanced-stage chronic diseases, including cardiovascular diseases (38.5%), cancer (34%), chronic respiratory diseases (10.3%), human immunodeficiency virus (5.7%), and diabetes (4.6%). Additionally, other conditions such as renal failure, chronic liver disease and neurodegenerative diseases like multiple sclerosis, Parkinson’s disease and dementia are increasingly recognized as requiring specialized PC services.

Timely identification of patients who may benefit from PC remains a significant challenge. Factors contributing to this difficulty include prognostic uncertainty, variability in disease trajectories, and the absence of standardized referral criteria [12]. In response to these challenges, different screening tools have been developed over the past two decades to help clinicians in recognizing patients with PC needs. These tools include traditional instruments based on clinical indicators, expert consensus, and scoring systems, as well as more recent models developed using artificial intelligence (AI) techniques. However, the characteristics, performance, and applicability of these tools vary considerably, and there is currently no consensus on which instruments are most effective or feasible across different care settings. Additionally, many tools lack robust validation or have only been tested in specific populations, limiting their generalizability. Nevertheless, global access to PC remains highly heterogeneous, with the majority of resources concentrated in high-income countries. Barriers including limited training of healthcare professionals, inadequate infrastructure, and insufficient awareness further hinder the timely integration of PC. In this context, standardized and validated screening tools could play a crucial role in bridging these gaps, ensuring early identification and equitable access to PC across diverse healthcare systems.

This systematic review aims to identify and critically appraise all screening tools developed for the early identification of patients with PC needs, encompassing both conventional instruments and models based on AI. This study examines the development methodology, clinical applicability, validation status, and key features of these tools to provide a comprehensive overview of existing approaches and guide future clinical and research initiatives.

## 2. Materials and Methods

This systematic review was conducted following the PRISMA 2020 (Preferred Reporting Items for Systematic Reviews and Meta-Analyses) guidelines [13] and the COSMOS-E (Conducting Systematic Reviews and Meta-analyses of Observational Studies of Etiology) recommendations [14]. Ethical approval was not required for this study as no human material or identifiable data were involved, and no human interventions were performed. For the same reason, obtaining informed consent was not necessary. The review protocol was registered in the International Prospective Register of Systematic Reviews (PROSPERO) under the registration number CRD420251031483 (available from https://www.crd.york.ac.uk/PROSPERO/view/CRD420251031483, accessed on 25 April 2025).

In line with the PRISMA framework, the research question was structured using the PICO format: P (Population): patients with advanced chronic illness; I (Intervention): screening tools; C (Comparator): not applicable; O (Outcomes): early identification of patients with potential PC needs.

### 2.1. Literature Search

We conducted a comprehensive literature search in PubMed, Web of Science, Cochrane Library, Scopus, EMBASE, and CINAHL to identify studies describing or evaluating tools designed for the early identification of patients with PC needs, including those developed using AI techniques. The search covered studies published from January 2000 to April 2025. The search was limited to studies published from 2000 onwards, as most tools for early identification of PC needs emerged after the development of modern PC frameworks and structured screening approaches at the turn of the century. The following search terms, including MeSH and free-text terms, were used: “Advanced Illness,” “Artificial Intelligence,” “Assessment Tools,” “External Validation,” “Identification,” “Machine Learning,” “Palliative Care Needs,” “Predictive Models,” and “Screening Tools”. The search strategy applied in each database combined these terms using Boolean operators (AND, OR). The search was limited to human studies, with no language restrictions. The full search strategy for PubMed is provided in the Supplementary Data.

### 2.2. Inclusion and Exclusion Criteria

We included studies that described or evaluated screening instruments developed to support the early identification of adult patients with PC needs. Eligible instruments targeted patients with advanced diseases, including cancer, heart disease, chronic respiratory disease, dementia, organ failure, frailty, and other severe conditions. We focused on tools designed to identify patients who may benefit from PC across diverse care settings (hospital, primary care, nursing home, or community), encompassing traditional clinical tools and those developed using AI techniques. Instruments designed explicitly for single conditions, such as cancer or heart failure, were also considered eligible. Given the rapid changes in patient conditions in these settings, we excluded screening instruments intended solely for use in intensive care units or emergency departments, as they limit their applicability in predicting longer-term PC needs. Additionally, we excluded tools primarily designed for use in the last days or weeks of life, as these are unsuitable for early identification of patients who may benefit from PC. Methodological exclusion criteria included review articles, duplicate publications, studies without usable data, small case series, and conference abstracts.

### 2.3. Study Selection

After removing duplicates, three reviewers (A.B.F., A.D.C.M., and G.G.M.) independently screened the titles and abstracts of all identified records to assess their eligibility based on the predefined inclusion and exclusion criteria. The full texts of potentially relevant articles were retrieved and evaluated in detail. Reviewer disagreements during the selection process were resolved through discussion and consensus. If consensus could not be reached, a fourth reviewer (A.F.M.) was consulted to make the final decision. To ensure consistency and reproducibility in the selection process, all reviewers used a standardized screening form to guide the application of the eligibility criteria.

### 2.4. Data Extraction

Data extraction was performed independently by three reviewers (A.B.F., A.D.C.M., and G.G.M.) using a standardized data collection form designed explicitly for this review. For each included study, the following information was extracted: first author, year of publication, country, study design, whether the tool was developed using traditional or AI-based techniques, study population, setting (hospital, primary care, nursing home, or community), characteristics of the screening tool, target disease or conditions, outcomes, and whether the tool had undergone external validation. We also collected information on whether the instrument incorporated multidimensional domains, such as psychological, social, or spiritual aspects. Performance metrics of the external validation studies were extracted when available, including sensitivity, specificity, positive predictive value (PPV), negative predictive value (NPV), and area under the receiver operating characteristic curve (AUC). When multiple versions of the same tool were available, data were extracted from the original version. The extracted data were synthesized to facilitate a qualitative comparison between the different tools. Any discrepancies in data extraction were resolved through discussion and consensus among the reviewers. When consensus could not be reached, disagreements were escalated to a fourth senior reviewer (A.F.M.), who acted as the final arbiter. Inter-rater agreement statistics were not calculated; however, methodological rigor was ensured through independent duplicate assessment, standardized data extraction forms, and structured consensus procedures, in accordance with PRISMA 2020 recommendations.

### 2.5. Outcomes

The objectives of this systematic review comprised identifying screening instruments designed to detect patients with early PC needs and assessing findings from external validation studies.

### 2.6. Risk of Bias Assessment

Four reviewers (A.B.F., A.D.C.M., A.F.M. and G.G.M.) independently assessed the methodological quality and risk of bias in the included studies. We assessed the methodological quality of the included studies using different tools tailored to each study design. For the development of instruments and questionnaires, we used the COSMIN checklist [15]. This tool evaluates content and structural validity, internal consistency, reliability, measurement error, responsiveness, and interpretability of the data. For observational studies, we applied the Newcastle–Ottawa Scale (NOS) [16]. We assessed the risk of bias in studies developing prediction models using the PROBAST tool [17]. This instrument evaluates four domains: participants, predictors, outcomes, and analysis. Each domain contains signaling questions that help identify potential sources of bias and concerns regarding applicability. This scale assesses three domains: participant selection, group comparability, and exposure or outcome ascertainment. For randomized clinical trials, we used the Cochrane Risk of Bias tool [18]. This instrument assesses bias across sequence generation, allocation concealment, blinding, incomplete outcome data, selective reporting, and other potential sources of bias. Each domain was categorized as having a low, high, or unclear risk of bias. Any discrepancies during the bias assessment process were resolved through discussion and consensus.

### 2.7. Data Synthesis and Analysis

The study selection process was systematically documented using a PRISMA 2020 flow diagram. Due to the heterogeneity in the development methodology, target populations, structure, and validation processes of the screening instruments, a narrative synthesis approach was employed. Data from the selected studies were systematically extracted and organized into structured tables to facilitate a comprehensive comparison. Screening tools were categorized into two main groups based on their development methodology: (1) conventional approaches and (2) AI-based techniques.

## 3. Results

### 3.1. Search Results

**Figure 1:**
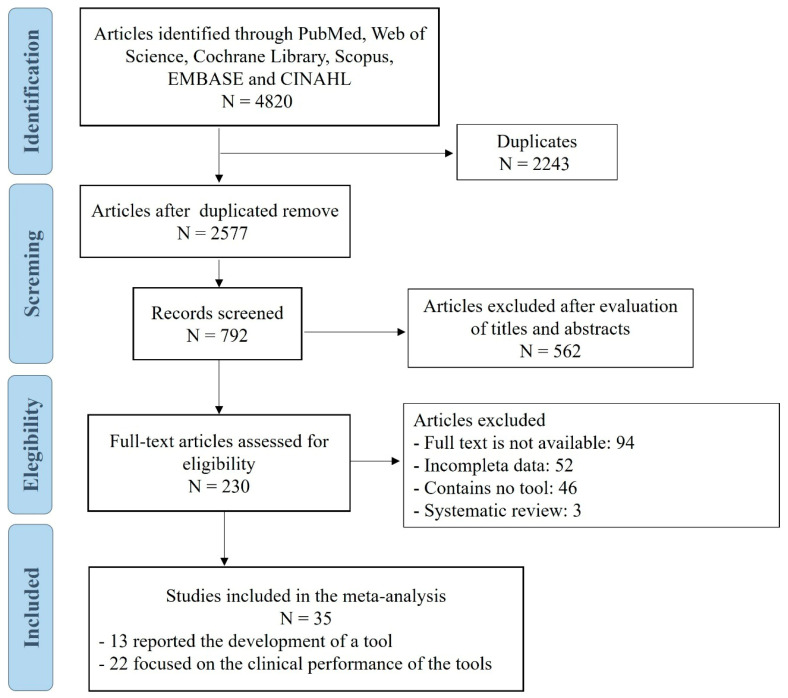
PRISMA 2020 flow diagram of study selection process.

A total of 4820 articles were initially retrieved. After removing 2243 duplicates, 2577 records remained. Of these, 792 were screened by title and abstract, resulting in the exclusion of 562 records. Following title and abstract screening, 230 articles were selected for full-text review. Finally, 35 studies were included: 13 reported the development of screening tools [19,20,21,22,23,24,25,26,27,28,29,30,31] and 22 focused on the external validation of these instruments [32,33,34,35,36,37,38,39,40,41,42,43,44,45,46,47,48,49,50,51,52,53] (Figure 1). Nine screening tools were developed using traditional methodologies, including the Gold Standards Framework Prognostic Indicator Guidance (GSF-PIG) [19], the Rainone [20], the Necesidades Paliativas CCOMS-ICO (NECPAL) [21], the RADboud indicators for PAlliative Care needs (RADPAC) [22], the Supportive and Palliative Care Indicators Tool (SPICT) [23], Anticipatory care in Primary care (AnticiPal) [24], Palliative Care Screening Tool (PCST) [25], the PROactive Palliative Care Identification Tool for patients with chronic obstructive pulmonary disease (COPD) (ProPal-COPD) [26], and PALLIA-10 [27], while four instruments were developed using AI techniques [28,29,30,31]. The Surprise Question (SQ) (“Would I be surprised if this patient died in the next 12 months?”) and the Double Surprise Question (DSQ) (when SQ is answered with ‘no’: “Would I be surprised if this patient will be still alive after 12 months?”) were identified as prognostic identification strategies and were analyzed separately but were not considered screening tools developed as standalone instruments. Among the instruments developed using traditional methods, seven were paper-based (GSF-PIG, NECPAL, RADPAC, SPICT, PCST, ProPal-COPD and PALLIA-10) and two were in electronic format (Rainone and AnticiPal).

### 3.2. Characteristics of the Screening Tools

The screening tools identified in this review differ in how they are intended for use in clinical practice. Some tools are designed for systematic screening, meaning that they are applied to all patients within a clearly defined population or clinical context. Other tools are not intended for universal screening, but rather to support clinician-led identification when clinical deterioration, complexity, or concern arises. Importantly, most tools do not provide explicit recommendations regarding when screening should be performed, how frequently it should be repeated, or which populations should be systematically assessed, which may hinder their consistent implementation across healthcare settings.

#### 3.2.1. Screening Tools Developed Using Traditional Methods

Table 1a shows the characteristics of the original studies that developed screening tools using traditional methods, while Table 1b summarizes the main features of the screening tools for the early identification of patients with PC needs.

The GSF-PIG was developed in the United Kingdom based on expert consensus and clinical experience [19]. It is applicable across all care settings and incorporates the SQ with general indicators of decline and disease-specific criteria. A negative answer to the SQ defines a positive result combined with at least one general or disease-specific indicator, such as frequent hospital admissions in patients with heart failure or loss of swallowing function in dementia. The Rainone tool was developed in the United States [20]. It targets individuals aged 75 years or older with congestive heart failure (CHF), COPD, dementia, cancer, or human immunodeficiency virus (HIV). Development methodology was based on retrospective analysis of electronic health records (EHRs) to determine factors most frequently associated with 12-month mortality. A positive case is defined by a negative response to the SQ and/or affirmative answers to any of the five additional items. The NECPAL CCOMS-ICO tool was developed in Spain, adapting elements from the GSF-PIG and SPICT instruments [21]. NECPAL combines the SQ with indicators of severity and disease progression for conditions including cancer, CHF, dementia, and COPD. A positive case is defined by a negative response to the SQ, along with the presence of at least one indicator. The RADPAC tool was developed in the Netherlands through a Delphi consensus study with general practitioners [22]. It is specifically designed for primary care. The tool includes disease-specific indicators for advanced CHF, COPD, and cancer stages. Notably, RADPAC uniquely incorporates patient-reported indicators, such as the patient’s perception that the end of life is approaching or a diminished ‘drive to live’, reflecting a subjective dimension rarely captured by other tools. The SPICT was developed in the United Kingdom through a structured process that included a literature review, peer review, and a prospective case-finding study [23]. The tool does not include the SQ. It is a checklist that combines general indicators of deteriorating health, such as declining functional status or increasing hospital admissions, with disease-specific indicators for conditions including cancer, organ failure, and dementia. It has been translated and culturally adapted into multiple languages. The AnticiPal tool was developed in the United Kingdom based on the SPICT criteria [24]. Its creation followed an iterative process involving design, implementation, and testing phases, combining clinical expertise with data from EHRs. AnticiPal is an automated search algorithm that identifies patients with potential PC needs based on Read Codes related to malignancy and serious chronic illnesses. The PCST was developed in the United States in the medical intensive care unit (MICU) setting [25]. In its original form, the tool consisted of a brief checklist of clinical and psychosocial indicators, and any positive item triggered a palliative care consultation within 24 h. The ProPal-COPD tool was developed in the Netherlands to identify patients with advanced COPD who may benefit from proactive PC. It was designed for systematic application in patients hospitalized for acute exacerbations of COPD and uses 1-year mortality prediction as a proxy for PC needs. The tool combines clinical judgment through the SQ with disease-specific indicators, including dyspnea severity, health status, lung function, body mass index, prior hospitalizations, and the presence of relevant comorbidities [26]. Finally, the PALLIA-10 tool was developed in France using a methodology that involved expert consensus and clinical practice observation [27]. It was designed for application in hospital settings. It consists of a checklist of 10 simple clinical indicators, including advanced chronic illnesses, repeated hospitalizations, unintentional weight loss, cognitive decline, and psychosocial or ethical concerns. In PALLIA-10, a positive screen is generally defined by a total score ≥ 3; however, a higher threshold (≥5) has been suggested as more specific and appropriate for identifying true PC needs.

#### 3.2.2. Screening Tools Developed Using Artificial Intelligence Techniques

Characteristics of the original studies describing the development of screening tools using AI methods are presented in Table 2. Avati et al. developed a mortality prediction model using a deep neural network trained on structured EHR data from the STRIDE clinical data warehouse at Stanford University [28]. The model was designed to identify hospitalized patients with a predicted risk of death within 3 to 12 months. The dataset included over 2 million patients, and training was conducted using a supervised learning approach. Wang et al. designed a deep learning model using neural networks with attention mechanisms (stacked LSTM layers) to predict 6-month, 1-year, and 2-year mortality in patients with dementia [29]. The model was trained on longitudinal clinical notes and demographic data from the Partners HealthCare System in Boston. Cary et al. employed a retrospective cohort design using Medicare administrative and clinical data from patients admitted to inpatient rehabilitation facilities following hip fracture [30]. They trained and compared logistic regression and multilayer perceptron (MLP) models to predict 30-day and 1-year mortality. The development process included extensive feature selection based on sociodemographic, clinical, and functional variables. Finally, Zhang et al. created a generalized machine learning pipeline (GMLP) to predict high-mortality-risk patients using administrative claims data from a regional health plan [31]. The pipeline included five stages: cohort creation, feature engineering, predictive modeling, scoring, and model maintenance. Development began with the application of a structured data pull strategy and inclusion/exclusion criteria based on PC eligibility. Feature engineering incorporated diagnosis codes, comorbidity indices, healthcare utilization costs and patterns, and demographic variables. None underwent external validation in independent populations or prospective clinical validation.

### 3.3. Risk of Bias Assessment of Screening Tool Derivation Studies

The methodological quality assessment is summarized in Tables S1–S3 (Supplementary Data files). For the checklist-based instruments (GSF-PIG, NECPAL, RADPAC, SPICT, AnticiPal, PCST, and PALLIA-10), content validity was adequate; however, other psychometric properties, such as reliability, internal consistency, and responsiveness, were not evaluated. The Rainone study received a score of 3 out of 9 stars on the NOS, and the ProPal-COPD tool achieved a total score of 8. The studies that developed ML-based prediction models have a high risk of bias in the analysis domain due to a lack of external validation and potential overfitting.

### 3.4. Performance of the Screening Tools in the External Validation Studies

Twenty one external validation studies evaluating four screening tools were identified (Table 3). These studies focused on predicting mortality or deterioration in various clinical settings, including acute hospitals, tertiary care centers, primary care, and specialized wards. The GSF-PIG was externally validated in three studies. Haga et al. assessed ambulatory patients with CHF NYHA III–IV, finding the highest sensitivity (83%) but the lowest specificity (22%) [32]. O’Callaghan et al. conducted a study in an acute hospital setting in New Zealand, reporting lower sensitivity (62.6%) but higher specificity (91.9%) [33]. Another study evaluated acutely hospitalized medical patients in South Africa (22% HIV and high prevalence of CHF), reporting a sensitivity of 74% and specificity of 85% [34]. The NECPAL tool has been evaluated in diverse settings. Gómez-Batiste et al. conducted a study in primary care and acute hospitals in Spain, reporting high sensitivity for mortality within 12–24 months (91.3% and 87.5%, respectively) [35]. Troncoso et al. adapted and validated the tool in Chilean primary care using a psychometric approach, reporting an AUC of 0.808 for predicting a positive response to the SQ [36]. A Calsina-Berna et al. conducted a cross-sectional prevalence study in a tertiary hospital in Spain, focusing on the identification and clinical characterization of inpatients with PC needs, without reporting metrics [37]. Another Spanish study compared the accuracy of the NECPAL to the PROFUND instrument in 146 nursing home residents (46.6% with dementia) for predicting assessed survival at 3, 6, 12, and 24 months [38]. The PROFUND instrument showed better predictive capacity in the short term (3 months), particularly among residents with dementia, whereas NECPAL performed better in the long term (24 months), especially among residents without dementia. Fisher et al. performed a cultural and linguistic adaptation of NECPAL for Israel (I-NECPAL), providing psychometric metrics (sensitivity 0.93, specificity 0.17, PPV 0.53, and NPV 0.71) related to the SQ [39]. Finally, a prospective study conducted in Italy evaluated the prognostic role of the NECPAL in 103 hospitalized older adults admitted for acute medical and surgical conditions [40]. One-year mortality was used as the primary outcome. The predictive accuracy of NECPAL was moderate (AUC 0.75). After removing the “≥2 comorbidities” item, NECPAL’s performance improved (AUC 0.78). The Taiwanese version of PCST (TW-PCST) was evaluated in four studies. A prospective study showed a good discriminative ability for 14-, 90-, and 180-day mortality in a large cohort of inpatients in an acute hospital setting in Taiwan with consistently high NPV [41]. At 90 days, a total-ABCD cut-off score of ≥2 provided the optimal balance, yielding the highest sensitivity (84.0%) together with an acceptable specificity (74.7%) and a high NPV (98.8%). Yen et al. conducted an extensive interventional cohort study to evaluate the TW-PCST for early identification of PC needs in hospitalized patients in Taipei [42]. A score ≥ 4 was independently associated with higher 90-day mortality (adjusted Odds Ratio 6.86). Yen et al. conducted another study for comparing the prognostic accuracy of the SQ and the TW-PCST for predicting 12-month mortality [43]. TW-PCST performed slightly better than SQ, as it showed higher specificity (92.0% vs. 90.6%) and AUC (0.689 vs. 0.680). In a third study conducted by Yen et al., the TW-PCST tool showed a high NPV for 6-month mortality when the score was <4 [44]. We identified seven studies that reported the clinical performance of SPICT. Among studies, one was a randomized controlled trial, and six were observational studies. In a retrospective cohort study for predicting one-year mortality, this tool showed a sensitivity of 84.1% and a specificity of 57.9% [45]. Notably, the authors analyzed the predictive performance of the two domains of the instrument separately: the general indicators of deteriorating health yielded an AUC of 0.758, and the clinical disease-specific indicators had an AUC of 0.748, with no significant difference between them (*p* = 0.638). Mitchell et al. conducted a randomized controlled trial in Australian general practice to compare the use of the SQ followed by the SPICT in SQ-positive cases with unguided clinical intuition among general practitioners in predicting 12-month mortality [46]. The combined SPICT + SQ approach yielded a sensitivity of 34.0% (95% CI 25.3–42.8) and a specificity of 95.8% (95% CI 93.0–98.6), with a PPV of 20.5% and an NPV of 97.9%. Although tool-based screening increased the identification of patients at risk compared with intuition alone, its relatively low sensitivity limited its ability to capture all patients who died during follow-up. In a prospective cohort study, 3640 patients aged 75 years or older were followed for 12 months to evaluate the performance of SQ versus SPICT, as applied through an automated electronic health record search [47]. The SQ identified 50% of deaths with a specificity of 99%, while the SPICT identified 58% with a specificity of 98%. When excluding 10 deaths classified as sudden, sensitivity increased to 69% for the SQ and 81% for the SPICT. A prospective multicenter study evaluated the prognostic value of the SPICT among 458 patients admitted to acute geriatric and cardiology units [48]. In acute geriatric units, the sensitivity reached 0.82 with an AUC of 0.822, and in cardiology units, the sensitivity was 0.69 with an AUC of 0.651. SPICT was adapted and psychometrically validated in a primary care setting in Chile, focusing on identifying patients with potential palliative care needs based on clinical judgment [49]. Performance assessment was based on internal consistency (Cronbach’s alpha = 0.86) and predictive validity, as indicated by Odds Ratios for specific clinical indicators, ranging from 30.81 to 71.11. A multicenter study evaluated the predictive performance of the SPICT tool in older adults receiving home-based medical care [50]. A total of 129 patients were prospectively assessed, and 6-month mortality was used as the outcome. The SPICT demonstrated acceptable discriminative ability, with a sensitivity of 0.80, specificity of 0.56, PPV of 0.57, NPV of 0.79, and an AUC of 0.78 when using the cut-off of four general indicators plus one clinical indicator. One study culturally adapted and validated the SPICT for use in mainland China [51]. The Mandarin version (SPICT-CH) was developed and tested in 212 hospitalized cancer patients. The psychometric evaluation demonstrated an acceptable content validity (Cronbach’s α 0.76). Another cross-sectional validation study also aimed to translate and culturally adapt the SPICT tool into Chinese and assess its preliminary performance in 388 hospitalized cancer patients [52]. Its screening performance was excellent, with an accuracy of 0.905. The ProPal-COPD tool underwent external validation in a multicenter prospective cohort study. The study included 523 patients hospitalized for an acute exacerbation of COPD across 10 hospitals, with 1-year all-cause mortality as the outcome measure [53]. During follow-up, 100 patients (19.1%) died within 12 months. The tool demonstrated moderate discriminative ability, with an AUC of 0.68.

**Figure 2:**
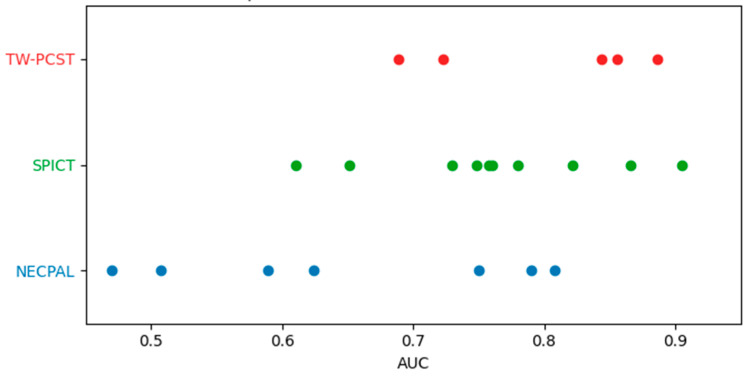
Distribution of reported AUC values across external validation studies of SPICT, NECPAL, and TW-PCST tools. Each dot represents an individual AUC value. Multiple AUC values across different prediction horizons were reported in the studies by Esteban-Burgos et al. [38] (NECPAL; 3-, 6-, 12-, and 24-month mortality) and Wang et al. [41] (TW-PCST; 14-, 90-, and 180-day mortality).

Figure 2 summarizes the distribution of reported AUC values across external validation studies of SPICT, NECPAL, and TW-PCST.

### 3.5. Risk of Bias Assessment of External Validation Studies

The risk of bias assessment of the external validation studies is shown in Tables S4 and S5 (Supplementary Data files). The studies showed heterogeneous quality. A considerable proportion of the observational validations of GSF-PIG and NECPAL were rated as having a high risk of bias, primarily due to limitations in participant selection (restricted or non-representative cohorts) and the use of non-objective outcomes (e.g., reliance on the SQ or clinical judgment). By contrast, the large Taiwanese cohorts validating the TW-PCST were consistently rated as having a low risk of bias, with a robust methodology. Similarly, the majority of SPICT validations were considered low-risk. The external validation of the ProPal-COPD tool had a high risk of bias, mainly due to methodological limitations in the analysis domain.

## 4. Discussion

To our knowledge, this is the first systematic review focusing on screening instruments for the early identification of patients with PC needs, including those that incorporate AI-based approaches.

This systematic review identified 12 screening tools for the early identification of PC needs. Eight tools were created using traditional methodologies, and four instruments employed artificial intelligence techniques. Overall, the methodological quality of these instruments was limited. Most of the identified tools use the prediction of death as a trigger for identifying people who are likely to have unmet PC needs. Patients with advanced progressive illness experience different trajectories of decline and typically have distinct needs across different stages of the disease. Therefore, the identification process should not be based solely on predicting mortality. It should also focus on anticipating their needs whenever they occur and predicting the rate and course of functional decline.

Importantly, only NECPAL and PCST (including its Taiwanese version) assessed psychological and spiritual care domains, but their approaches differ substantially. NECPAL integrates them as a structured domain with several dedicated items, whereas the PCST acknowledges such issues only within the “other criteria” category. These represent a small proportion of the total score and do not constitute a structured domain, reflecting the tool’s primarily biomedical and functional focus. PC is based on a holistic approach that addresses the patient’s physical, psychological, social, and spiritual needs. According to the WHO definition of PC, which encompasses physical, psychological, social, and spiritual dimensions, serious illness impacts multiple interrelated domains, resulting in complex patient needs. Thus, screening instruments should comprehensively assess these domains to effectively identify unmet needs. Psychological and spiritual distress are prevalent in patients with advanced diseases and can exacerbate physical suffering, while psychological and social needs become increasingly prominent as the end of life approaches [54,55,56].

Several previous reviews have summarized tools for the early identification of palliative care needs [57,58,59]. However, the present review extends prior work by incorporating recently developed tools, including disease-specific instruments and AI-based models, and by systematically evaluating predictive performance and risk of bias using contemporary methodological frameworks. In addition, this review provides a comparative perspective between traditional screening approaches and emerging data-driven models, highlighting shared limitations and future research directions. A relevant but largely unexplored limitation identified in this review is the underutilization of rich longitudinal clinical data beyond diagnostic and mortality outcomes. In particular, pharmacy records represent a valuable yet insufficiently exploited data source for early identification of PC needs. Changes in medication patterns—such as the initiation or escalation of potent opioids, repeated adjustments in diuretic therapy in advanced heart failure, or increasing polypharmacy—may act as sensitive proxies for symptom burden, functional decline, and escalating care complexity. Future screening strategies, including AI-based models and refined traditional tools, should incorporate pharmacometrics trajectories as indirect signals to support earlier, more needs-oriented identification.

All AI-based models identified in this review were designed to predict mortality rather than multidimensional PC needs. While mortality remains a commonly used proxy, this approach risks overlooking psychological, social, and spiritual dimensions that are central to PC. Future AI-based approaches may offer opportunities to integrate multidimensional data sources, such as patient-reported outcomes, functional status, and social vulnerability, moving beyond purely prognostic models towards tools better aligned with the holistic goals of PC.

A notable gap identified in this review is the scarcity of external validation studies for the screening tools. Despite their intended clinical utility, most instruments lack robust evidence supporting their accuracy and reliability across diverse settings and populations.

In the context of external validation, TW-PCST demonstrated the most consistent performance across multiple time points in the hospital setting. Unlike NECPAL, which exhibited high sensitivity but low specificity, TW-PCST maintained a more balanced profile, minimizing false positives. In contrast, GSF-PIG showed greater variability in its performance across different patient populations. Tools that include the SQ, such as NECPAL and GSF-PIG, may introduce subjectivity, potentially increasing the rate of false positives. This subjectivity can be particularly pronounced in non-cancer populations, where disease trajectories are less predictable and clinical judgment may be less reliable [60,61]. In contrast, tools that do not rely on subjective prognostication, like TW-PCST, may offer more objective results. Beyond identification accuracy, the clinical value of screening tools lies in their capacity to trigger timely and meaningful clinical action. A positive screening result should not be viewed as an endpoint, but rather as a catalyst for predefined interdisciplinary interventions. For example, the output of tools such as SPICT or an automated AI-based alert could be embedded into clinical workflows to prompt predefined interdisciplinary responses, such as a pharmacist-led medication review in the presence of escalating symptom burden, followed by a structured, team-based goals-of-care conversation when unmet needs or prognostic uncertainty are identified. Explicitly linking screening outputs to actionable care pathways may enhance the translation of early identification into tangible palliative care interventions and improve real-world clinical impact.

Integrating AI into PC is an opportunity to overcome the limitations of traditional models. Embedding AI-based screening tools within electronic health records may enhance their practical value by enabling automated, continuous identification of at-risk patients, potentially reducing clinician workload and variability associated with manual screening. In contrast, traditional screening methods require active clinician input and time for application, which may hinder scalability and consistent use in routine practice. Hybrid models combining automated AI-based screening with clinician assessment may therefore represent a promising strategy to improve efficiency while preserving clinical judgment. AI approaches can leverage extensive datasets to identify complex patterns that extend beyond clinical indicators, thereby anticipating decline trajectories. However, despite their potential, the studies included in our review reveal substantial limitations. Notably, all four AI-based studies lacked external validation. Without robust external testing, the generalizability of these models remains speculative. Furthermore, while AI models can theoretically capture multidimensional needs, including psychosocial and spiritual distress, current implementations focus predominantly on mortality prediction. Therefore, the added benefit of AI remains challenging to assess in this clinical setting.

Building on these findings, an ideal future screening tool should integrate multiple complementary dimensions. Such a framework would combine the clinical intuition and needs-oriented perspective embedded in tools like NECPAL with the predictive capacity of AI-based models, while systematically incorporating structured assessments of psychological, social, and spiritual domains. This integrated approach would allow screening tools to move beyond single-domain identification and better reflect the complexity of palliative care needs in routine clinical practice.

Why is it so complex to implement early identification processes for patients with PC needs? In light of our systematic review, one of the primary reasons is the persistence of a biomedical model that prioritizes mortality and prognosis in decision-making. We believe that incorporating the biopsychosocial model—which considers suffering as well as psychological, social, and spiritual dimensions—and advancing towards hybrid models that integrate both approaches would significantly enhance anticipation and early detection. Additional challenges further hinder this process, including insufficient professional training and limited knowledge of the biopsychosocial model, the lack of time in daily clinical practice, the absence of universal protocols, and, finally, the limitations imposed by the terminology currently used. In this regard, we propose to replace the term “palliative care” with “integrated early palliative approach” Although efforts have been made in recent years to broaden the acceptance of the term “palliative care” to promote its earlier and more widespread use, the expected impact in clinical practice has not been achieved. Therefore, a terminological shift is suggested. Nevertheless, caution is warranted. These patients should remain within the palliative perspective and approach to avoid creating parallel categories that, rather than integrating, might fragment care. These measures would help ensure greater equity in the inclusion of patients in palliative care.

Our study has some limitations. First, the systematic review included only a few high-quality studies. Therefore, the results should be interpreted with caution. Meta-analysis was not feasible due to substantial heterogeneity across studies, including differences in target populations, outcomes, and prediction horizons, and variability in cut-off thresholds used across validation studies. Second, we conducted a comprehensive literature search to capture all the relevant papers. Still, given the nature of this topic, some papers may have been missed. Third, there is currently no consensus on how to measure the accuracy of a screening tool for the early identification of patients with PC needs. In this context, specialist PC assessment may be a more appropriate reference standard for identifying PC needs than mortality outcomes, which are often used as surrogates but do not fully reflect the clinical complexity underlying PC identification.

## 5. Conclusions

This systematic review identified 13 studies on screening tools for detecting advanced illness and potential PC needs. Their evaluation is limited by the lack of a standardized comparator, which constrains conclusions about real-world utility. Most tools rely on mortality prediction, despite the multidimensional nature of palliative needs. Overall accuracy is moderate, reinforcing the need for clinical judgment. The findings underscore the urgency of standardized, multidimensional screening approaches linked to actionable care pathways. Future research should prioritize the development and external validation of tools that integrate clinical, functional, psychological, social, and spiritual domains into routine care. An integrated early palliative approach may enable earlier identification, timely interdisciplinary intervention, and more equitable access across disease trajectories.

## Figures and Tables

**Table 1:** (**a**) Characteristics of the original studies describing the development of screening tools using traditional methods for the early identification of patients with palliative care needs. (**b**) Characteristics of the screening tools developed using conventional methodologies for the early identification of patients with palliative care needs.

(**a**)									
**Author**	**Tool**	**Year**	**Country**	**Other Versions**	**Development Methodology**	**Sample Size**	**Age**	**Population**	**Setting**
Thomas et al. [19]	GSF-PIG	2004	United Kingdom	Italy	Development based on clinical expert consensus and literature review	NA (theoretical tool development)	NA	Patients with advanced progressive illness	Primary care, hospital, nursing homes, and community
Rainone et al. [20]	Rainone	2007	United States	No	Quality improvement project combining EHR screening and clinician judgment	1154 patients evaluated (from 1993 identified); 299 deemed eligible for PC referral	≥75	Patients with advanced chronic illnesses (CHF, COPD, dementia, cancer, HIV)	Primary care
Gómez-Batiste et al. [21]	NECPAL	2012	Spain	Argentina Chile Brazil Portugal	Adaptation and extension of GSF-PIG and SPICT indicators through expert consensus, cultural adaptation, and preliminary prospective validation	50,000 screened; 1162 patients with advanced chronic conditions; 684 were NECPAL+	81.73 ± 12.01 (NECPAL+)	General population with advanced chronic conditions	Primary care, hospital, nursing homes, and community
Thoonsen et al. [22]	RADPAC	2012	Netherlands	No	Development based on literature review, focus groups with GPs, and Delphi consensus process	NA (theoretical tool development)	NA	Patients with CHF, COPD, or cancer	Primary care
Highet et al. [23]	SPICT	2014	United Kingdom	Japanese Spanish German Taiwan	Development based on literature review, peer review, prospective case investigation	Pilot case review (130 cases evaluated)	NR	Patients with advanced progressive conditions and health deterioration	Primary care, hospital, and community
Mason et al. [24]	AnticiPal	2015	United Kingdom	No	Iterative software development approach	NR	NR	Patients with advanced illnesses	Primary care
Hurst et al. [25]	PCST	2017	United States	Taiwan	Checklist based on clinical criteria; adapted and applied in a quasi-experimental design	223	59.94 ± 15.72	Patients admitted to the medical intensive care unit	Hospital
Duenk et al. [26]	ProPal-COPD	2017	Netherlands	No	Prospective cohort study	155	67.0 ± 9.4	Patients hospitalized for acute exacerbation of COPD	Hospital
Molin et al. [27]	PALLIA-10	2019	France	No	Prospective multicenter study conducted in comprehensive cancer centers	841 included, 687 palliative patients analyzed	64.8 (median)	Cancer patients	Hospital
(**b**)									
**Tool**	**Target Patients**		**Number of Indicators**	**Inclusion of SQ**	**Inclusion of Psychological and Spiritual Concerns**	**Tool-Positive Criteria**		**Outcome**	**External** **Validation**
GSF-PIG [19]	Patients with advanced illness or deteriorating health status		NA	Yes	No	Positive if the SQ response is “No” and at least one general or disease-specific indicator is present		Risk of mortality within months, weeks, or days	Yes
Rainone [20]	Patients with advanced chronic illnesses (CHF, COPD, dementia, cancer, HIV)		6	Yes	No	Clinical consideration prompted by a “No” response to the SQ and/or affirmative responses to any of the five additional items. No formal scoring cutoff		Risk of clinical deterioration or death within 12 months	No
NECPAL [21]	Patients with advanced chronic conditions		59	Yes	Yes	Positive if the response to the SQ is “No” and at least one additional clinical indicator is present		Prediction of 38-, 17.2- and 3.6-month mortality	Yes
RADPAC [22]	Patients with COPD, CHF, or cancer		21	No	No	Presence of at least one disease-specific indicator suggesting palliative care needs; no predefined scoring threshold		Risk of clinical deterioration	No
SPICT [23]	Patients with advanced conditions and deteriorating health		17	No	No	SPICT (2019 version): No formal cut-off; identification is based on clinical judgment informed by the presence of general and/or clinical indicators SPICT-J (Japanese version): positive if ≥2 general indicators or ≥1 clinical indicator are present SPICT-ES (Spanish version): positive if ≥2 general indicators and ≥1 clinical indicator are present		Risk of clinical deterioration	Yes
AnticiPal [24]	Patients with advanced illness identified through electronic health records		NA (automated search algorithm based on Read Codes)	No	No	Patient identified if at least one inclusion criterion (malignancy codes, single Read Codes, or combinations of codes) is met, and no exclusion criteria are present		Prediction of 12-month mortality	No
PCST [25] *	Hospitalized patients in the medical intensive care unit with advanced disease or poor prognosis		6	No	Yes	≥1 items		Increased frequency of palliative care consultations and reduced time to consultation	Yes ^†^
ProPal-COPD [26]	Patients hospitalized for acute exacerbation of advanced COPD		7	Yes	No	Positive SQ combined with a multivariable risk score above the predefined threshold		1-year mortality	Yes
PALLIA-10 [27]	Hospitalized cancer patients		10	No	No	PALLIA-10 score ≥ 3 indicates the need for palliative care referral (although a higher threshold, ≥5, has been suggested for greater specificity)		Identification of patients with short-term mortality risk	No

Abbreviations: GSF-PIG, Gold Standards Framework Prognostic Indicator Guidance; NECPAL CCOMS-ICO, Necesidades Paliativas Centro Colaborador de la Organización Mundial de la Salud—Institut Català d’Oncologia; RADPAC, RADboud indicators for PAlliative Care needs; SPICT, Supportive and Palliative Care Indicators Tool; AnticiPal, Anticipatory care in Primary care; PCST, Palliative Care Screening Tool; ProPal-COPD, PROactive Palliative Care Identification Tool for patients with chronic obstructive pulmonary disease; PALLIA-10, Palliative Care Screening Checklist with 10 Indicators; CHF, congestive heart failure; COPD, chronic obstructive pulmonary disease; EHR, electronic health record; GPs, general practitioners; HIV, human immunodeficiency virus; NA, not applicable; NR, not reported; PC, palliative care; SQ, Surprise Question. * The Taiwanese version of the PCST (TW-PCST) includes 26 clinical indicators structured across four domains: (A) basic disease process, (B) comorbidities, (C) functional status (ECOG Performance Status), and (D) other clinical indicators such as repeated hospitalizations, prolonged intensive care unit stay, and uncontrolled psychosocial or spiritual issues. The TW-PCST has broadened its scope to include general hospitalized patients. ^†^ The TW-PCST version.

**Table 2:** Characteristics of screening tools developed using artificial intelligence for the early identification of patients with palliative care needs.

Tool	Year	Country	ML Methodology	Setting	Target Patients	Validation
Avati et al. [28]	2018	USA	Deep Neural Network	Hospital/Inpatient	Hospitalized adults for early identification of PC needs (prediction of mortality within 3–12 months)	Internal. Validation was performed using a hold-out test set from the same Stanford STRIDE dataset (train/test split)
Wang et al. [29]	2019	USA	Recurrent Neural Network (LSTM)	Healthcare System/Dementia Patients	Patients with dementia for early identification of PC needs (mortality prediction at 6 months, 1 year, and 2 years)	Internal. The dataset was split into a training set (90%) and a held-out test set (10%) from the same health system (Partners HealthCare).
Cary et al. [30]	2021	USA	Multilayer Perceptron (MLP) and Logistic Regression	Inpatient Rehabilitation Facilities	Older adults (>65) with hip fracture for early identification of PC needs (30-day and 1-year mortality)	Internal. Validation was conducted through stratified 10-fold cross-validation within the same cohort of post-hip fracture rehabilitation patients.
Zhang et al. [31]	2021	USA	AdaBoost Algorithm within a Generalized ML Pipeline	Health Plan Population	General high-risk patients from administrative claims data for early identification of PC needs	Internal. Validation was performed within data from the same regional health plan.

Abbreviations: ML, machine learning; MLP, multilayer perceptron; LSTM, long short-term memory; PC, palliative care.

**Table 3:** External validation studies of tools for the early identification of patients with palliative care needs.

Tool	Author (Year)	Country	Outcome	Sample Size	Age, Mean	Setting	Sensitivity	Specificity	NPV	PPV	AUC
GSF-PIG	Haga et al. (2012) [32]	United Kingdom	12-month mortality	138	77	Ambulatory patients with CHF NYHA class III–IV	83	22	5	33	NR
	O’Callaghan et al. (2014) [33]	New Zealand	12-month mortality	501	70	Acute hospital	62.6	91.9	90	67.7	NR
	Raubenheimer et al. (2019) [34]	South Africa	12-month mortality	822	45 (median)	Hospitalized patients (22% HIV and high prevalence of CHF)	74	85	93	56	NR
NECPAL	Gómez-Batiste et al. (2017) [35]	Spain	Mortality at 12 and 24 months	1057	81	Primary care and acute hospital	12-month mortality: 91.3 24-month mortality: 87.5	12-month mortality: 32.9 24-month mortality: 35	12-month mortality: 91 24-month mortality: 81.7	12-month mortality: 33.5 24-month mortality: 45.8	NR
	Troncoso et al. (2021) [36]	Chile	Psychometric validation (predicting positive SQ)	118	71.92 ± 10.18	Primary care	NR	NR	NR	NR	0.808
	Calsina-Berna et al. (2022) [37]	Spain	Prevalence and clinical characterization of patients with PC needs	227	81 (median)	Tertiary hospital (internal medicine and geriatric wards)	NR	NR	NR	NR	NR
	Esteban-Burgos et al. (2023) [38]	Spain	Mortality at 3, 6, 12 and 24 months	146	84.6 ± 8.9	Nursing homes	NR	NR	NR	NR	3-month mortality: NECPAL: 0.507 PROFUND: 0.641 6-month mortality: NECPAL: 0.470 PROFUND: 0.616 12-month mortality: NECPAL: 0.589 PROFUND: 0.564 24-month mortality: NECPAL: 0.624 PROFUND: 0.442
	Fisher et al. (2024) [39]	Israel	Psychometric validation (identification of PC needs)	376	78	Hospital (internal medicine ward)	93	17	71	53	0.79
	Spannella et al. (2024) [40]	Italy	12-month mortality	103	86.8 ± 7.2	Hospitalized older population	NR	NR	NR	NR	0.75
TW-PCST	Wang et al. (2019) [41]	Taiwan	Mortality at 14, 90, and 180 days	21,596	57.6 ± 24.5	Acute hospital	14-day mortality: 80.9 90-day mortality: 84 180-day mortality: 81.6	14-day mortality: 85.1 90-day mortality: 74.7 180-day mortality: 74.5	14-day mortality: 99.5 90-day mortality: 98.8 180-day mortality: 97.8	14-day mortality: 10 90-day mortality: 16.4 180-day mortality: 22.8	14-day mortality: 0.886 90-day mortality: 0.856 180-day mortality: 0.844
	Yen et al. (2020) [42]	Taiwan	90-day mortality	47,153	61.7 ± 19.3	Hospitalized patients in Taipei city	NR	NR	NR	NR	6.86 *
	Yen et al. (2022) [43]	Taiwan	12-month mortality	21,109	62.8 ± 19.3	Hospitalized patients in Taipei city	45.8	92	94.9	34.1	0.689
	Yen et al. (2022) [44]	Taiwan	6-month mortality	111,483	60.9	Hospitalized patients in Taipei city	49.6	94.9	98.1	26.6	0.723
SPICT	De Bock et al. (2018) [45]	Belgium	12-month mortality	435	84 (median)	Acute geriatric unit	84.1	57.9	NR	NR	General indicators: 0.758 Clinical indicators: 0.748
	Mitchell et al. (2018) [46] ^§^	Australia	12-month mortality	1525	77.9	Primary care	34	95.8	20.5	97.9	NR
	van Wijmen et al. (2020) [47]	The Netherlands	12-month mortality	3640	≥75	Primary care	SPICT 58 SQ 50	SPICT 98 SQ 99	NR	NR	NR
	Piers et al. (2021) [48]	Belgium	12-month mortality	458	≥75	Acute geriatric and cardiology units	AUG 0.82 CU 0.69	AUG 0.49 CU 0.67	AUG 89.3 CU 86.8	AUG 33.9 CU 33.7	AUG 0.822 CU 0.651
	Farfán-Zuñiga et al. (2022) [49]	Chile	Psychometric validation (identification of PC needs)	292	79.4	Primary care	NR	NR	NR	NR	0.866 ^†^
	Liao et al. (2024) [50]	Taiwan	6-month mortality	129	82.4 ± 12	Older adults receiving home-based medical care	80 (4 general + 1 clinical indicator)	56 (4 general + 1 clinical indicator)	79 (4 general + 1 clinical indicator)	57 (4 general + 1 clinical indicator)	General indicators: 0.73 Clinical indicators: 0.61 4 general + 1 clinical indicator: 0.78
	Xie et al. (2025) [51]	China	Psychometric validation (identification of PC needs)	212	56 (20–81)	Hospitalized oncological patients	NR	NR	NR	NR	0.76 ^†^
	Huang et al. (2025) [52]	China	Screening of PC needs	388	57 (18–89)	Hospitalized cancer patients	80	94	92	84	0.905
ProPal-COPD	Broese et al. (2022) [53]	Netherlands	1-year all-cause mortality	523	70.0 ± 9.1	Hospitalized patients with COPD	55	74	NR	NR	0.68

* Adjusted Odds Ratio with a score ≥ 4. ^†^ Cronbach’s α. ^§^ SPICT + SQ. Abbreviations: AUG, acute geriatric units; CHF, chronic heart failure; COPD, chronic obstructive pulmonary disease; CU, cardiology units; GSF-PIG, Gold Standards Framework Prognostic Indicator Guidance; HIV, human immunodeficiency virus; NECPAL CCOMS-ICO, Necesidades Paliativas Centro Colaborador de la Organización Mundial de la Salud—Institut Català d’Oncologia; PC, palliative care; SPICT, Supportive and Palliative Care Indicators Tool; SQ, Surprise Question; TW-PCST, Taiwanese Palliative Care Screening Tool; ProPal-COPD, PROactive Palliative Care Identification Tool for patients with chronic obstructive pulmonary disease; AUC, area under the receiver operating characteristic curve; NPV, negative predictive value; NR, not reported; NYHA, New York Heart Association; PPV, positive predictive value.

## Data Availability

The data supporting this study’s findings are available from the corresponding author upon reasonable request. Restrictions apply to the availability of these data, which were used under license for this systematic review.

## Supplementary Materials

The following supporting information can be downloaded at https://www.mdpi.com/article/10.3390/jcm15030919/s1: Table S1: Quality of the studies describing the development of screening tools according to the COSMIN criteria; Table S2: Quality of the studies describing the development of screening tools using the Newcastle–Ottawa Scale; Table S3: Quality of the studies describing the development of screening tools according to the PROBAST instrument; Table S4: Risk of bias in external validation studies according to PROBAST criteria; Table S5: Risk of bias in external validation studies according to the Cochrane Risk of Bias tool; Table S6: PRISMA Checklist.

## Abbreviations

The following abbreviations are used in this manuscript:

AI	Artificial Intelligence
AUC	Area Under the Receiver Operating Characteristic Curve
CHF	Congestive Heart Failure
CI	Confidence Interval
COPD	Chronic Obstructive Pulmonary Disease
COSMIN	COnsensus-based Standards for the selection of health Measurement INstruments
COSMOS-E	Conducting Systematic Reviews and Meta-analyses of Observational Studies of Etiology
DSQ	Double Surprise Question
EHR	Electronic Health Record
GSF-PIG	Gold Standards Framework Prognostic Indicator Guidance
HIV	Human Immunodeficiency Virus
ML	Machine Learning
NA	Not Applicable
NOS	Newcastle–Ottawa Scale
NPV	Negative Predictive Value
NR	Not Reported
NYHA	New York Heart Association
OR	Odds Ratio
PC	Palliative Care
PCST	Palliative Care Screening Tool
PPV	Positive Predictive Value
ProPal-COPD	PROactive Palliative Care Identification Tool for patients with Chronic Obstructive Pulmonary Disease
RADPAC	RADboud indicators for PAlliative Care needs
SPICT	Supportive and Palliative Care Indicators Tool
SPICT-CH	Chinese version of the Supportive and Palliative Care Indicators Tool
SQ	Surprise Question
TW-PCST	Taiwanese version of the Palliative Care Screening Tool
WHO	World Health Organization
