# Supplementary material for: Screening Tools for the Early Identification of Palliative Care Needs in Patients with Advanced Chronic Conditions: An Updated Systematic Review
Source: J Clin Med. 2026 Jan 23;15(3):919. doi: 10.3390/jcm15030919 (PMC12898093; doi:10.3390/jcm15030919)
Supplement: Supplementary file 1 [file jcm-15-00919-s001.zip › jcm-4079735-supplementary-V2.pdf]

Full PubMed search strategy
Database: PubMed/MEDLINE
Date of last search: 30 April 2025
Timeframe: 1 January 2000 – 30 April 2025
Language restrictions: None
Population: Humans

Search strategy (verbatim)

```
(
  "Palliative Care"[MeSH Terms]
  OR "Palliative care"[Title/Abstract]
  OR "Supportive care"[Title/Abstract]
)
AND
(
  "Needs Assessment"[MeSH Terms]
  OR "Palliative care needs"[Title/Abstract]
  OR "unmet needs"[Title/Abstract]
)
AND
(
  "Screening"[Title/Abstract]
  OR "Screening tool"[Title/Abstract]
  OR "Identification tool"[Title/Abstract]
  OR "Assessment tool"[Title/Abstract]
  OR "Predictive model"[Title/Abstract]
  OR "Risk stratification"[Title/Abstract]
  OR "Prognostic"[Title/Abstract]
  OR "Artificial intelligence"[Title/Abstract]
  OR "Machine learning"[Title/Abstract]
)
AND
(
  "Chronic Disease"[MeSH Terms]
  OR "Advanced illness"[Title/Abstract]
  OR "Advanced chronic disease"[Title/Abstract]
  OR "Life-limiting illness"[Title/Abstract]
)
```

Table S1. Quality of the studies describing the development of screening tools according to the COSMIN criteria.

Tool	Content validity	Structural validity	Reliability	Construct validity	Responsiveness	Overall quality
GSF-PIG [19]	Adequate	Not applicable	Not assessed	Limited	Not assessed	Low–moderate
NECPAL [21]	Adequate	Not applicable	Not assessed	Moderate	Not assessed	Moderate
RADPAC [22]	Adequate	Not applicable	Not assessed	Limited	Not assessed	Low–moderate
SPICT [23]	Adequate	Not applicable	Not assessed	Moderate	Not assessed	Moderate
AnticiPal [24]	Adequate	Not applicable	Not assessed	Limited	Not assessed	Low–moderate
PCST [25]	Adequate	Not applicable	Not assessed	Strong	Partial	High
PALLIA-10 [27]	Adequate	Not applicable	Not assessed	Moderate	Not assessed	Moderate

Abbreviations: GSF-PIG, Gold Standards Framework Prognostic Indicator Guidance; NECPAL CCOMS-ICO, Necesidades Paliativas Centro Colaborador de la Organización Mundial de la Salud - Institut Català d'Oncologia; RADPAC, RADboud indicators for PAlliative Care needs; SPICT, Supportive and Palliative Care Indicators Tool; AnticiPal, Anticipatory care in Primary care; PCST, Palliative Care Screening Tool; PALLIA-10, Palliative Care Screening Checklist with 10 Indicators.

Table S2. Quality of the studies describing the development of screening tools using the Newcastle–Ottawa Scale.

Tool	Selection	Comparability	Outcome	Total	Risk of bias
Rainone [20]	☆☆	—	☆	3/9	High
ProPal-COPD [26]	☆☆☆☆	☆	☆☆☆	8/9	Low

Table S3. Quality of the studies describing the development of screening tools according to the PROBAST instrument.

Study	Participants	Predictors	Outcome	Analysis	Risk of bias
Avati et al. [28]	Low	Low–Moderate	Low	High	High
Cary et al. [29]	Low	Low	Low	High	High
Wang et al. [30]	Low	Low	Low	High	High
Zhang et al. [31]	Low	Low–Moderate	Low	High	High

Table S4. Risk of bias in external validation studies according to PROBAST criteria.

Study	Participants	Predictors	Outcome	Analysis	Risk of Bias
Haga et al. [32]	High	Low	Low	High	High
O'Callaghan et al. [33]	Low	Low	Low	Low	Low
Raubenheimer et al. [34]	High	Low	Low	Low	High
Gómez-Batiste et al. [35]	Low	Low	Low	Low	Low
Troncoso et al. [36]	High	Low	High	High	High
Calsina-Berna et al. [37]	High	Low	High	High	High
Esteban-Burgos et al [38]	High	Low	Low	High	High
Fisher et al. [39]	High	Low	High	High	High
Spannella et al [40]	High	Low	Low	High	High
Wang et al. [41]	Low	Low	Low	Low	Low
Yen et al. [42]	Low	Low	Low	Low	Low
Yen et al. [43]	Low	Low	Low	Low	Low
Yen et al. [44]	Low	Low	Low	Low	Low
De Bock et al. [45]	Low	Low	Low	Low	Low
van Wijmen et al [47]	Low	Low	Low	Low	Low
Piers et al [48]	Low	Low	Low	Low	Low
Farfán-Zuñiga et al. [49]	Low	Low	High	High	High
Liao et al [50]	Low	Low	Low	Low	Low
Xie et al. [51]	High	Low	High	High	High
Huang et al [52]	Low	Low	Low	Low	Low
Broese et al. [53]	Low	Low	Low	High	High

Table S5. Risk of bias in external validation studies according to the Cochrane Risk of Bias tool.

Study	Randomization process	Deviations from intended interventions	Missing outcome data	Outcome measurement	Selection of the reported result	Risk of Bias
Mitchell et al. [46]	Low	Low	Low	Low	Low	Low
